# Bioassay for Endothelial Damage Mediators Retrieved by Hemoadsorption

**DOI:** 10.1038/s41598-019-50517-1

**Published:** 2019-10-10

**Authors:** Maximilian Denzinger, Ludger Staendker, Keno Ehlers, Julian M. Schneider, Tanja Schulz, Tabea Hein, Sebastian Wiese, Annika Roecker, Ruediger Gross, Jan Münch, Hendrik Bracht, Eberhard Barth, Manfred Weiss, Michael Georgieff, E. Marion Schneider

**Affiliations:** 1 Division of Experimental Anesthesiology, University Hospital Ulm, Albert-Einstein-Allee 23, 89081 Ulm, Germany; 2 Core Facility Functional Peptidomics, Ulm University, Albert-Einstein-Allee 47, 89081 Ulm, Germany; 3 Institute of Pharmacology and Toxicology, Ulm University, Albert-Einstein-Allee 11, 89081 Ulm, Germany; 4 Institute of Molecular Virology, Ulm University Medical Center, Meyerhofstrasse 1, 89081 Ulm, Germany; 5 Department of Anesthesiology, University Hospital Ulm, Albert-Einstein-Allee 23, 89081 Ulm, Germany

**Keywords:** Blood proteins, Sepsis, Sepsis, Preclinical research, Preclinical research

## Abstract

Hemoadsorption devices are used to treat septic shock by adsorbing inflammatory cytokines and as yet incompletely defined danger and pathogen associated molecular patterns. In an ideal case, hemoadsorption results in immediate recovery of microvascular endothelial cells’ (mEC) function and rapid recovery from catecholamine-dependency and septic shock. We here tested a single device, which consists of polystyrene-divinylbenzene core particles of 450 μm diameter with a high affinity for hydrophobic compounds. The current study aimed at the proof of concept that endothelial-specific damage mediators are adsorbed and can be recovered from hemoadsorption devices. Because of excellent clinical experience, we tested protein fractions released from a hemoadsorber in a novel endothelial bioassay. Video-based, long-term imaging of mEC proliferation and cell death were evaluated and combined with apoptosis and ATP measurements. Out of a total of 39 fractions recovered from column fractionation, we identified 3 fractions that caused i) inhibition of mEC proliferation, ii) increased cell death and iii) induction of apoptosis in mEC. When adding these 3 fractions to mEC, their ATP contents were reduced. These fractions contained proteins of approximately 15 kDa, and high amounts of nucleic acid, which was at least in part oxidized. The efficacy for endothelial cell damage prevention by hemoadsorption can be addressed by a novel endothelial bioassay and long-term video observation procedures. Protein fractionation of the hemoadsorption devices used is feasible to study and define endothelial damage ligands on a molecular level. The results suggest a significant effect by circulating nucleic acids – bound to an as yet undefined protein, which may constitute a major danger-associated molecular pattern (DAMP) in the exacerbation of inflammation when patients experience septic shock. Hemoadsorption devices may thus limit endothelial damage, through the binding of nucleic acid-bearing aggregates and thus contribute to improved endothelial barrier function.

## Introduction

To date, the complexity of pathologies in septic shock may explain why therapeutic approaches targeting a specific mediator have largely failed. The criteria to define septic shock include organ dysfunction^1^. One reason for the functional deterioration of vital organs is the loss of barrier function by endothelial cells^2^. Out of a panel of innovative therapeutic options, blood purification therapies are promising strategies to maintain barrier function^3–22^. To verify efficacies of blood filtrations and adsorption, bioassays measuring endothelial-cell integrity as a read-out parameter should be preferred.

On the cellular level, immune interaction of activated leukocytes with endothelial lining cells guides the pro-inflammatory phenotype linked to disseminated coagulation and destruction of the endothelial barrier. Endothelial barriers are key for an appropriate oxygen and nutrient supply of vital organs, as reviewed by Opal *et al*.^23^. Accordingly, when the immune system of the host attempts to combat the invading pathogens, substantial collateral damage will lead to impaired barrier function in endothelial but also epithelial cells. Interestingly, the damage response of destroyed tissues follows largely identical pathways as pathogen-linked inflammation^24^. There is ample evidence that organ damage requires a panel of mediators and the same may be true for tissue regeneration and reconstitution. Consequently, this may be one reason for the failure of a multitude of clinical trials in sepsis and septic shock where only selected mediators where targeted^23^.

Despite the likelihood of such complicated molecular networks in driving organ failure in sepsis and septic shock, we here attempted to identify protein fractions retrieved by CytoSorb adsorption that lead to endothelial damage in the absence of white blood cells. Due to a lack of clinical evidence for beneficial hemoadsorption approaches in septic shock, being restricted to case reports, case series and small studies, and larger controlled clinical trials need to be initiated^22^. Experimental evidence is also scarce such as the work by David *et al*., using human umbilical vein endothelial cells (HUVECs) and septic serum incubation to demonstrate disruption of the HUVECs cell layers, whereas post hemoadsorption serum samples displayed no detrimental effect on HUVECs^8^. The work by Gruda and colleagues implies that CytoSorb treatment not only removes cytokines, but also danger- and pathogen- associated molecular patterns (DAMPs, PAMPs), which might explain the significant advantage of this treatment option^10^. Their results performed *in vitro* show that not only cytokines, but also DAMPs and PAMPs, need to be removed to improve clinical outcomes. Hydrophobicity and molecular weight-dependent sieve function may be key for clinically successful treatment. Despite the likely involvement of multiple mediators in the pathology of septic shock, with a molecular weight ranging between 10–60 kDa, which may explain the experimental and clinical evidence for the resolution of septic shock by this hemoadsorption device^7,9,25^, we here approached the identification and enrichment of defined protein fractions based on their capacity to influence endothelial cell viability.

We removed proteins adsorbed to CytoSorb beads and subjected the eluant to protein fractionation. From each individual CytoSorb adsorber, a total of 39 protein fractions were obtained and tested by appropriate bioassays using microvascular endothelial cells (mEC). Exemplarily, protein fractions of a single adsorber were tested for their effect on proliferation, death, apoptosis and adenosine triphosphate (ATP) contents of brain-derived endothelial cells. These procedures identified three fractions that led to endothelial damage, whereas the remaining fractions were largely inactive. The active fractions appear to contain a defined protein entity as well as nucleic acids, which were in part oxidized. The active fractions are now ready for further characterization to stratify patients into groups which could possibly benefit from hemoadsorption therapy.

## Patients, Materials and Methods

### Patients treated with hemoadsorber CytoSorb

Patients were treated with CytoSorb according to the process instructions for hemoadsorption of our intensive care unit (ICU), G1. Analysis of patients’ immune phenotypes and plasma biomarkers and the biochemical analysis of material adsorbed to Cytosorb columns were performed according to the Helsinki declaration and the ethics vote #150/16 approved by the Ethics Committee of Ulm University, and all samples were collected with the informed written consent of the patients and volunteers.

The study design was as follows: Patients were enrolled to our study when admitted to the ICU within 48 hours after sepsis onset and presenting with high plasma interleukin (IL)-6 concentrations (>500 pg/ml) and/or kidney failure. When a patient was enrolled and planned to be treated with CytoSorb hemoadsorption therapy, one blood sample was taken before the treatment. During the treatment, infection markers were routinely checked. After 24 hours of treatment, another blood sample was taken. The CytoSorb adsorber cartridge was immediately processed (see below). Cytokine measurements and leukocyte antigen expression analysis were performed before and after CytoSorb hemoadsorption treatment.

We here report representative results on the biochemical preparation of a single hemoadsorption device performed in a 76-year-old female patient after surgery for an abdominal aneurysm through Crawford access and clamping of visceral and renal arteries. Sepsis was diagnosed, according to Singer and colleagues (2016), by the presence of catecholamine-dependent septic shock, hemodynamics and a lactate concentration >2 mg/dl following the Sepsis 3 criteria on day 3 after surgery (catecholamines were administered at 0.4 µg/kg Body Weight (BW)/min, lactate was 4.9 mg/dl), and inflammatory variables were highly elevated, including C-reactive protein (CRP): 307.6 mg/l; procalcitonin (PCT): 5.68 µg/l; IL-6: 4944 pg/ml; and IL-8: 308 pg/ml; as was lactate dehydrogenase (266 U/l). The patient benefitted from CytoSorb treatment in that the catecholamine dosage was decreased to 0.05 µg/kgBW/min and most of the other inflammatory parameters declined, including CRP (235.5 mg/l), PCT (4.3 µg/l), IL-6 (107.4 pg/ml) and IL-8 (63.6 pg/ml), whereas lactate remained elevated (5.2 µg/l). The flow chart below summarizes the clinical and experimental procedure for CytoSorb-based hemoadsorption and the experimental set up.

**Figure 1:**
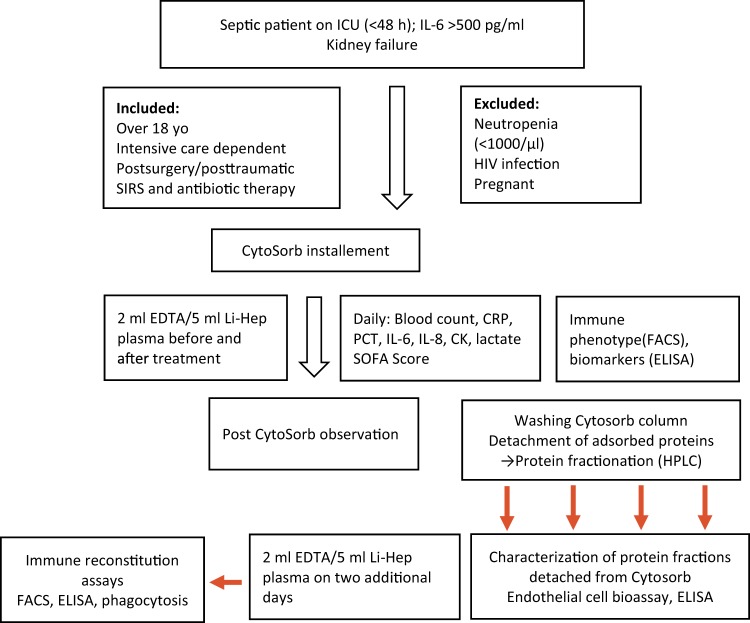
Flowchart of our Working Procedure. This schematic describes the process instructions for hemoadsorption on ICU and Clinical Study to identify mediators removed by hemoadsorption and documentation of a potential improvement of the patient.

The flowchart in Fig. 1 describes the process instructions for hemoadsorption on ICU performed for our Clinical Study to identify mediators removed by hemoadsorption and a potential improvement of the patient.

### Endothelial bioassay

Microvascular endothelial cells (mEC) were established from embryonal rat brain cultures. Following spontaneous transformation, mEC were cloned, and each of the clones presented with some morphological but not phenotypic differences. One clone, termed C2-6, established a confluent cell layer of cobble-stone morphology and spontaneously formed vessel-like structures (Supplementary Video V1). The parent cell line C2 was previously shown to vascularize glioblastoma tumors in an appropriate tumor model^26^. C2-6 mEC expressed typical surface markers, including *Ulex europaeus* lectin >30%, Dil-Ac-LDL >70%, EN7/44 (endothelial and proliferation marker) >80% and factor VIIIa >40%. This mEC cell line also responded to leukemia inhibitory factor (LIF), oncostatin M (OSM) and basic fibroblast growth factor (bFGF) as well as astrocyte supernatants with significantly increased proliferation. An astrocyte-conditioned medium also induced CD36 expression (data not shown). The parent clone of C2, was previously used to study LPS-inducible cell death^27^.

Cells were grown in RPMI1640 supplemented with 25 mM HEPES, antibiotics, 10% fetal calf serum (FCS superior, very low endotoxin (Biochrom.de, Germany)) and GlutaMAX (ThermoFisher.com, USA) at 37 °C and 5% CO_2_. When confluency was reached, the cells were detached using 0.05% trypsin/ethylenediaminetetraacetic acid (EDTA), and seeded in new flasks.

### CytoSorb hemoadsorption cartridge preparation after treatment and fractionation

Following the patients’ treatment according to the manufacturer’s instructions, the CytoSorb cartridge was washed free from cellular blood components as well as non-adsorbed plasma proteins using phosphate-buffered saline (PBS) containing 0.005% EDTA until the buffer outflow was visibly clear and no blood components remained in the cartridge. Details and images are provided in Supplementary Fig S1. Scanning electron microscopy was applied to determine residual protein aggregates and cellular components bound to the CytoSorb beads surfaces (Supplementary Fig. S2, note that there is free space on individual beads after treatment). The protein material adsorbed to the beads of a CytoSorb cartridge was processed. Protein separation results of a single, randomly selected cartridge run in a 76-year-old female patient is shown in the present investigation. Protein detachment from CytoSorb beads was performed using 50% acetonitrile in water for 1 hour, 1/10 dilution of the clear extract and a subsequent ultrafiltration step (cut-off: 30 kDa) to remove albumin. Following chromatography, the proteins were lyophilized and resuspended in 2 ml resuspension solution (1% trifluoroacetic acid in H_2_O and 5% of buffer containing 80% acetonitrile and 0.1% trifluoroacetic acid in H_2_O), followed by 10 minutes of centrifugation at 4,200 rpm. Supernatants were collected and sterilized by filtration with 0.22 µm pore size. High-performance liquid chromatography was performed with a flow rate of 1.3 ml/min, and protein collection began after 5 minutes for 40 minutes. This separation yielded 39 protein fractions for further testing in a newly established mEC bioassay (see Supplementary Fig. S3).

### IncuCyteZOOM-based video microscopy and mEC bioassay

#### Microscopy imaging

For long-term microscopy imaging investigations, the incubator microscope IncuCyteZOOM (sartorius.com, USA) was installed at 37 °C, 5% CO_2_ and 100% humidity. C2-6 mEC were seeded at 1.5 × 10^4^ cells per well into transparent, flat-bottomed 96-well plates (thermofischer.com) in 100 µl RPMI supplemented with 10% FCS, Glutamax and antibiotics. Following mEC attachment to the plate within 12 hours, showing excellent morphology, the test compounds were added. L-homocysteine (Hcy) (sigmaaldrich.com) was reduced with twice the amount of dithiothreitol in 50 mM TRIS buffer at pH 8 for 30 minutes at 37 °C, and served as a positive control for mEC-specific damage responses. Cell death was monitored by propidium iodide (PI) uptake (added to a final concentration 2 µg/ml), using the red fluorescent filter and an acquisition time of 800 ms. To determine proliferation rates, phase-contrast images were recorded using the IncuCyteZOOM software.

#### Quantification of ATP

To determine the cytoplasmic ATP contents, adherent cells of individual 96-well cultures were trypsinized and resuspended in 90 µl to be transferred into a white chemiluminescence plate and incubated at 37 °C under 5% CO_2_ over night to ensure homogeneous cell-layer formation. The lyophilized fractions of material detached from the CytoSorb cartridge were reconstituted with PBS buffer, and 10 μl of these were added to the individual 96-well cavities with a final volume of 100 µl. ATP concentrations were determined using the CellTiter-Glo® Luminescent Cell Viability Assay (promega.com). Luminescence was detected using the Glomax instrument (promega.com) at different time intervals.

#### Annexin V staining

To detect apoptosis and pyroptosis, Annexin V and PI staining were performed using a protocol slightly modified from Hang Xi *et al*.^28^.

Endothelial cells (2 × 10^5^/ml) were seeded into 3.5 cm ∅ petri dishes (thermofisher.com). As a positive control for endothelial-cell damage, 1 mM reduced Hcy (10 µg/ml) was added. CytoSorb fractions were tested at 10% (v/v).

Following incubation for 24 hours, adherent and floating mEC were collected and subjected to Annexin V and PI staining followed by flow cytometric analysis. A final cell count of 5 × 10^4^ cells was recorded for each culture condition.

#### Scanning electron microscopy

Endothelial cell clone C2-6 was seeded onto glass coverslips and fixed with 2.5% glutardialdehyde (sigma.com), washed and dehydrated by critical-point desiccation. Specimens were processed by carbon/gold spattering and microscopically examined at 10 keV using a Hitachi scanning microscope.

#### Data analysis

Statistical analysis of IncuCyteZOOM data files, two-way ANOVA was applied using GraphPad Prism, vs.7. Values of p < 0.05 and lower were defined as statistically significant. For quantitative evaluation of flow cytometric data, FlowJo® vs. histograms were used.

## Results

### Confluency and cell death in the presence of CytoSorb-derived protein fractions

**Figure 2:**
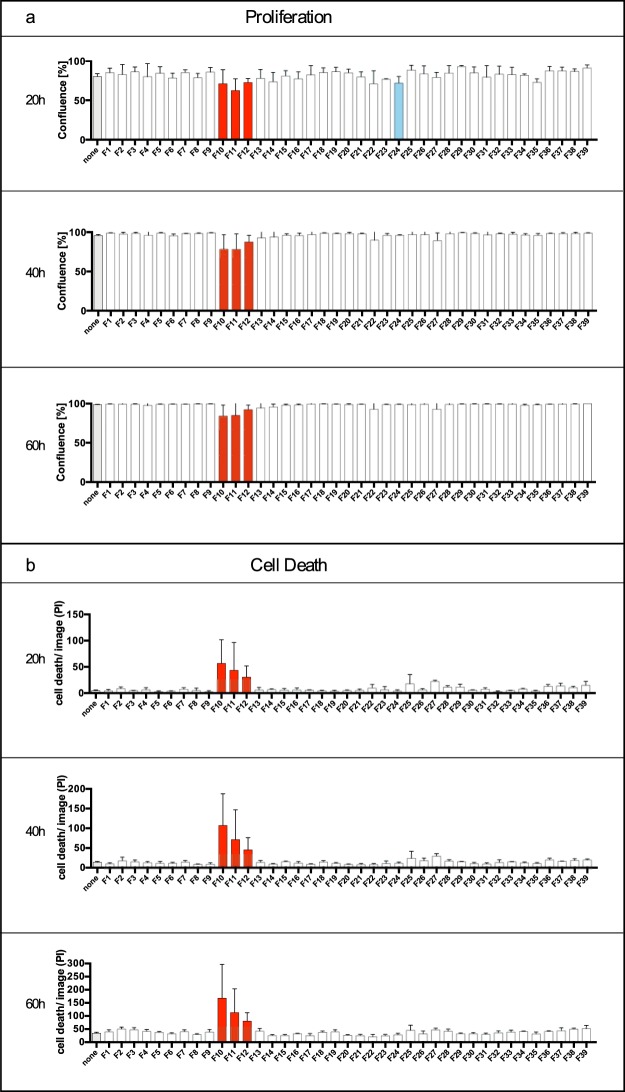
Pattern of CytoSorb protein fractions on microvascular endothelial cells (mEC) growth and cell death. mEC were cultured in the absence (none) and presence of 1:10 diluted protein fractions (F) for 60 hours in total, with individual measurements at every hour: Confluency was determined by optical densities using the IncuCyteZOOM. Protein fractions resulting in significantly diminished confluence rates are shown as red (F10–F12) and blue (F24) columns following two-way ANOVA (multiple comparison) (**a**). Protein fractions resulting in cell death of cultured mEC were determined by adding 2 ng/ml propidium iodide to the culture medium; counting of red fluorescent nuclei was accomplished by IncuCyteZOOM videomicroscopy and red object count quantification. Fractions leading to significantly increased cell death are shown by a red column (**b**). Relevant time points of 20 hours, 40 hours and 60 hours are shown of the continuous observation. Significances were determined by two-way ANOVA (multiple comparison). All values are given as means and standard deviation (of triplicate cultures) for statistically relevant protein fractions. Images were obtained with the IncuCyteZOOM using a 20× objective, phase contrast and fluorescence detection (565–605 nm excitation, 625–705 nm emission).

We here identified protein fractions derived from CytoSorb adsorbers with a significant effect on the viability and proliferation of brain-derived mEC. For this, we used a newly established bioassay providing a high sensitivity to mediators involved in endothelial damage of affected organs in sepsis and septic shock. Figure 2 shows the influence of the protein fractions F1–F39 on endothelial cell proliferation following continuous, hourly observation and measurements for 60 hours. F10–F12 where highly active in the downmodulation of endothelial cell confluence (Fig. 2a) as well as in endothelial cell death (Fig. 2b) over the total time of 60 hours. Differences between non-treated and CytoSorb fraction-treated growth curves (red-colored columns) were observed at 24 hours (p < 0.001), 10 h for F11 (p < 0.0001), and 25–28 hours, and 35–39 hours for F12 (p < 0.05) (for details see Supplementary Fig. S4).

In addition, F24 displayed some influence on cell proliferation at an early time point (blue-colored column, after 13–22 hours) (Fig. 2a; the confluency at other timepoints (40 hours, 60 hours) were not significantly different (for details see Supplementary. Fig. S4).

Video microscopy was also applied to determine cell death in the presence and absence of CytoSorb-derived protein fractions. Generally, the spontaneous endothelial cell death level was 8% (30–50 cells per image). Fractions F10, F11 and F12 resulted in a significantly increased cell death level of 29%, 20% and 14%, respectively, as determined after 60 hours (p < 0.0001, p < 0.0001, p < 0.01). The first time point of significant differences in the presence of F10–F12 was at 16 hours of observation (p < 0.05). Neither fraction F24 nor the remaining protein fractions induced any cell death throughout this experiment. When compared with the non-treated control, the earliest significant difference in viability induced by the addition of CytoSorb fractions was documented at 32 hours for F10, at 16 hours for F11 and at 30 hours for F12 (p < 0.05). Significances for F10 and F11 were p < 0.0001 and for F12 p < 0.01 when calculated from the first instance of statistical significance until the observational endpoint by multiple comparison two-way analysis of variance (ANOVA). These results suggest that the concentration of the unknown endothelial damage factor(s) may be highest in F11. Note that the effects of F24 on cell viability and confluency were comparatively marginal (Fig. 2). This fact may suggest a different pathophysiology by F24 compared with F10–F12.

In summary, four different protein fractions were classified as “active” (F10–F12, F24) for their significant inhibitory effect on mEC proliferation. The remaining fractions had no effect on mEC proliferation and did not increase cell death. The same procedure was performed with hemoadsorbed material from two other patients, one of whom benefitted from CytoSorb treatment (data not shown). A total of 46 protein fractions were obtained from the responding patient, two of which: F26-F27 impaired mEC proliferation and increased cell death. By contrast, none of the 44 protein fractions obtained from the hemoadsorbed material of the non-responding patient affected mEC confluency or viability (data not shown). The reasons for these differences are likely related to the individual elution profile of the column fractionation and await further investigations.

### Apoptosis detection

**Figure 3:**
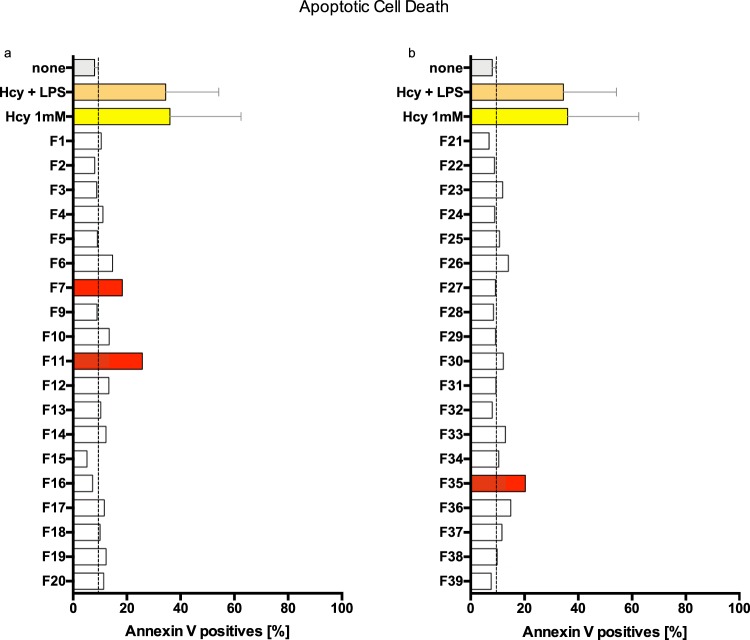
Modulation of Annexin V expression by microvascular endothelial cells (mEC). mEC were cultivated in petri dishes (2 × 10^5^ cells per dish) and incubated for 24 hours with reduced L-homocysteine (Hcy, 1 mM) with or without lipopolysaccharide (LPS, 10 µg/ml) or with CytoSorb-derived protein fractions at a final dilution of 1:10. Cells were detached using trypsin and stained with Annexin V and propidium iodide (PI). Flow cytometry was performed at a final cell count of 5 × 10^4^ per assay. Annexin V-positive cells are shown. For control (none) and positive control (Hcy, Hcy + LPS) means of four replicates and standard deviation (SD) are displayed, SD of the control is shown (dotted line). Protein fractions F7 and F11 induced apoptosis, with F11 resulting in more than three times more Annexin V-positive cells when compared to the control (25.80% vs. 7.99%) **(a)**. Protein fraction F35 also induced apoptosis in more than twice as many cells as in the control (20.30% vs. 7.99%) **(b)**.

Apoptosis-induced cell death was addressed by flow cytometric quantification of Annexin V binding and PI staining. According to the results presented in Fig. 3, the red-colored fractions displayed increased Annexin V binding when compared to the 8% (6.7–9.6%) Annexin V-positive cells monitored in the non-treated mEC. Incubation with CytoSorb fractions for 24 hours resulted in increased apoptotic mEC by F7 (18.3%), F11 (25.8%) and F35 (20.3%). Referring to the mEC viability assay, F11 (Fig. 2) was likely to induce apoptosis as confirmed by increased Annexin V binding (Fig. 3). Moreover, fractions F7 and F35 also induced increased Annexin V binding (Fig. 3), but had no effect on mEC viability (Fig. 2). The remaining fractions tested did not induce Annexin V binding on mEC following 24 hours of incubation. In summary, F10–F12 affected viability directly, F11 influenced viability by apoptosis induction and F7 and F35 induced Annexin V binding but without subsequent cell death (Fig. 2). Because of its well-known detrimental effect on mEC viability, reduced Hcy served as a positive control in our experiments^29–33^.

### Homocysteine-mediated apoptosis in mEC

**Figure 4:**
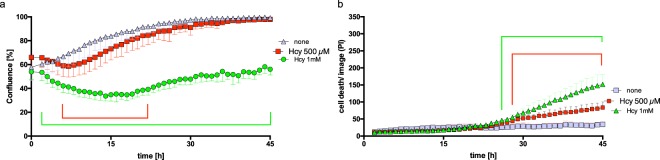
Microvascular endothelial cells (mEC) are sensitive to the known toxic agent L-homocysteine (Hcy). Endothelial cell growth in the absence (control) and presence of 0.5 mM or 1 mM reduced Hcy was monitored for 45 hours (x-axis). For fluorescent detection of cell death, PI was used (0.05 mg/ml). Confluency was measured by optical analysis with the IncuCyteZOOM software using a confluence mask and given as percent (y-axis). Adding 1 mM reduced Hcy resulted in decreased (from 0 hours 53.9% to 14 hours 33.8%) confluency and inhibited cell growth to 56% of the control (45 hours). Compared to the control, we found a dose-dependent decrease in mEC confluency, by 8% at 7 hours for 0.5 mM and by 20% at 14 hours for 1 mM Hcy. The difference in confluency compared to control was statistically significant between 1 hour and 45 hours (green bracket) (multiple comparison two-way ANOVA, p < 0.0001) **(a**). Cell death is displayed as the red object count (dead cells)/image (y-axis)). Reduced Hcy at 1 mM showed significantly more cell death/image compared to the control at 26 hours to 45 hours (green bracket) (multiple comparison two-way ANOVA, p < 0.0001). Values are given as means, while standard deviation (triplicates) is shown for the statistically relevant protein fractions **(b**).

**Figure 5:**
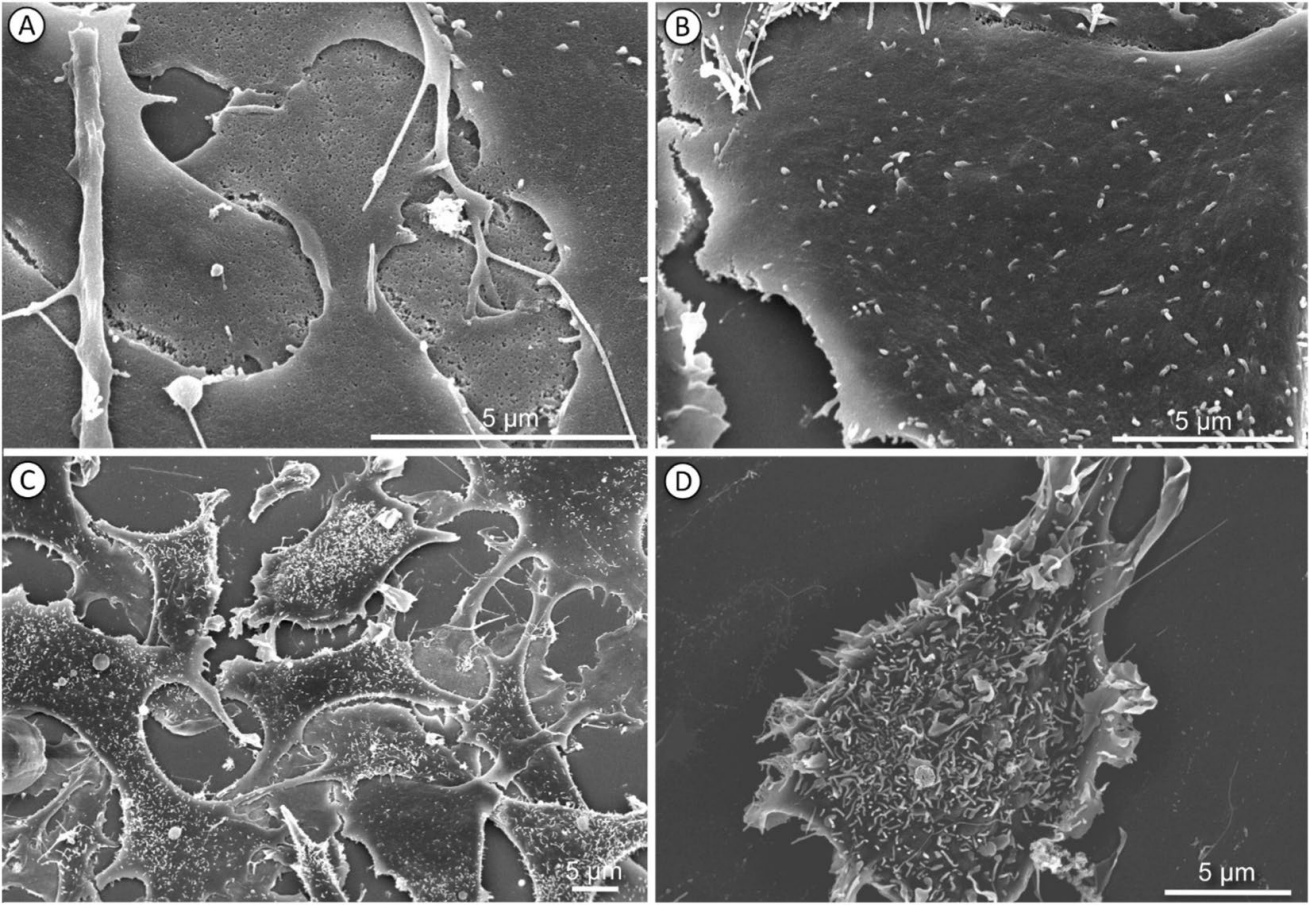
Morphology of microvascular endothelial cells (mEC) C2-6 in the absence and presence of homocysteine. Scanning electron micrograph of mEC cultured in the absence (**a**,**b**) and in the presence of L-homocysteine for 24 hours (**c**,**d**). Note the impaired cell-to-cell contact formation and high density of stress-associated microvilli in Hcy-treated mEC.

Figure 4 demonstrates the influence by exogenous Hcy on mEC by long-term video microscopy. Cell death is displayed as the red object count for 0.5 mM and 1 mM reduced Hcy, and was significant after 27 hours and 26 hours of incubation to the end of observation, respectively (multiple comparison two-way ANOVA, p < 0.0001). Distinct from the effect of CytoSorb fractions, Hcy-treated mEC appeared to recover after 30 hours, resulting in a U-shaped curved shape of the confluence measurements (Fig. 4a). To further address the effects of Hcy, we performed scanning electron microscopy of constitutive and Hcy-treated mEC (Fig. 5). Non-treated mEC had a largely smooth surface, whereas Hcy-treated mEC demonstrated extended formation of microvilli on their surface, in addition to impaired confluency and significantly affected cell-to-cell contact.

### Protein fractions affecting ATP content

**Figure 6:**
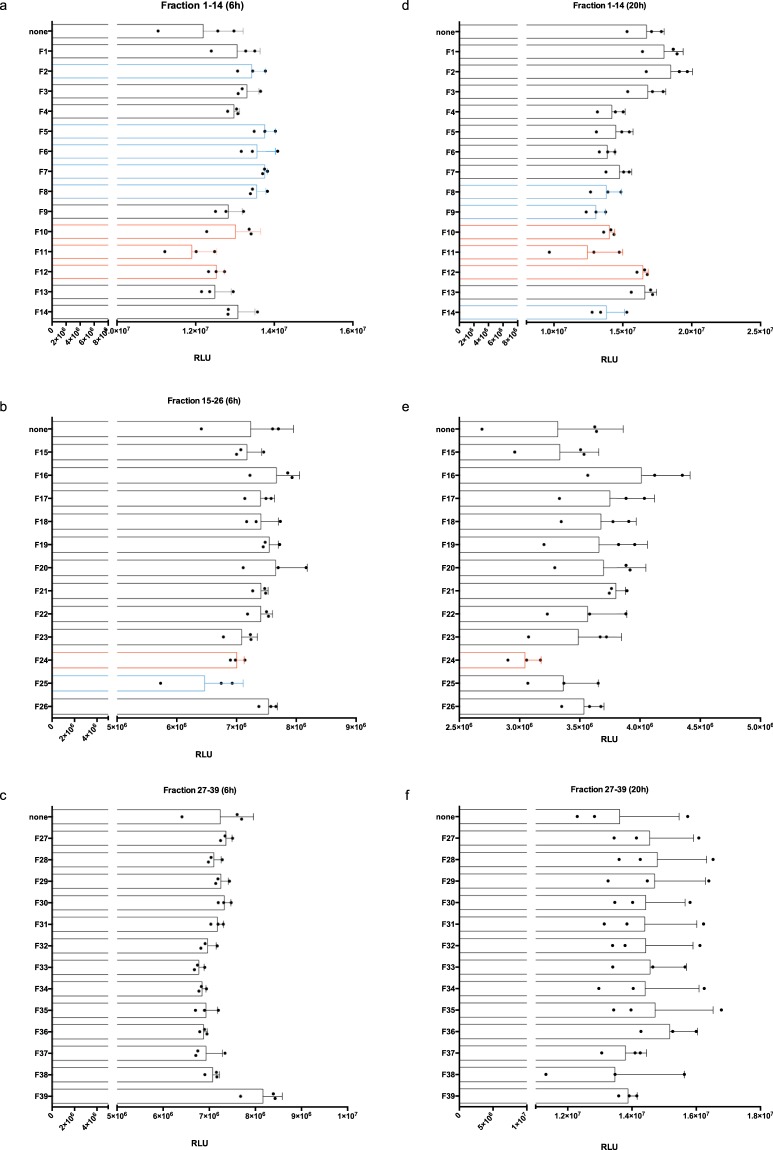
ATP contents in microvascular endothelial cells (mEC) after 6 and 20 hours of incubation with CytoSorb-derived protein fractions. ATP contents in the absence (none) and presence of protein fractions (F, diluted 1:10 v/v) were recorded for 6 hours (left column; **a–c**) and 20 hours (right column; **d–f**). Cells were lysed after 6 and 20 hours and intracellular ATP contents were determined as relative luminescent units (RLU) (ATPCellTiter Glo, Promega). After 6 hours of incubation, some protein fractions showed significantly higher ATP levels when compared to the control: F2 (p < 0.05); F5 (p < 0.01); F6 (p < 0.05); F7 (p < 0.01); F8 (p < 0.05); F39 (p < 0.01) (Dunnett´s multiple comparison test); whereas protein fraction F25 diminished ATP contents (p < 0.05) when compared to the control (**a**–**c**). After 20 hours of incubation, the following protein fractions decreased ATP contents in mEC: F8 (p < 0.05); F9 (p < 0.01); F11 (p < 0.01); F14 (p < 0.05) (Dunnett´s multiple comparison test) (d–f). Values are given as means and standard deviation of triplicates. The results were representative for two independent experiments. All fractions with a different trend of either upregulation or downmodulation of ATP contents at 6 hours and 20 hours of incubation were labeled by blue border lined bars and fractions consistently impairing ATP contents were labeled by red-bordered columns (F10–F12, F24).

To determine the potential influence on metabolism, we tested CytoSorb-derived protein fractions on the ATP content. ATP in mEC was quantified after 6 and 20 hours of incubation with all protein fractions (Fig. 6). After 6 hours, we found somewhat elevated ATP levels when treating mEC with F2, F5, F6, F7, F8, F25 or F39 (Fig. 5a–c), but this ATP increase was not mirrored by increased proliferation (cf. Fig. 2). After 20 hours, we found significantly decreased ATP contents in F8, F9, F11 and F14 co-incubation experiments (Fig. 6d–f). Additionally, F24 downregulated the ATP content, but this difference was not highly significant when compared to the non-treated mEC control culture (p < 0.05) (Fig. 6). Fractions with a different trend of either upregulation or downmodulation of ATP contents at 6 hours vs. 20 hours of incubation are labeled by blue border lines of the columns and fractions consistently impairing ATP contents are labeled by red-bordered columns (i. e. F10–F12, F24).

### DAMP ligands in hemoadsorption-derived protein fractions

**Figure 7:**
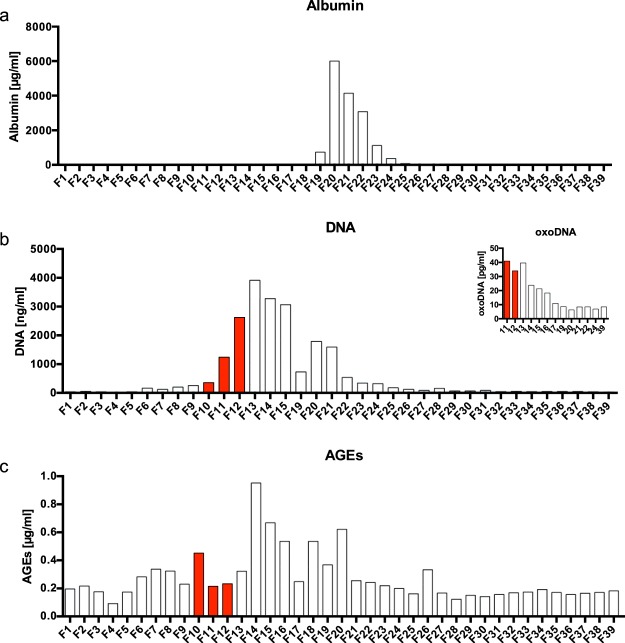
Characterization of CytoSorb-derived protein fractions. Albumin contents (ng/ml, y-axis) in given fractions (x-axis) using a high-sensitive ELISA (Immulite1000®). Fractions F1–F18 and F26–F39 were below the detection limit (4 µg/ml). The highest amount of albumin was found in F20 (>6000 µg/ml). **(a)** Amounts of deoxyribonucleic acids (DNA) were determined by spectrophotometry at 260/280 nm (NanoDrop). The highest amounts of DNA were found in F13 (3915.6 ng/ml) **(b**) the insert shows the contents of oxidized DNA (8-hydroxy-2′-deoxyguanosine from DNA, 8-hydroxyguanosine from RNA and 8-hydroxyguanine from DNA or RNA); results were obtained by ELISA (Cayman Chemicals.com); the highest concentration was found in F11 (40.97 pg/ml). Advanced glycation end products (AGEs) were quantified by ELISA (mybiosource.com); the highest amount was found in F14 (0.95 µg/ml); means of duplicates’ analyses are shown. **(c)** Red-filled bars were fractions which consistently influenced endothelial viability, ATP contents, and apoptosis.

The contribution of cell signaling by CytoSorb-derived fractions was investigated by the quantification of protein-fraction contents, including albumin, but also a selection of DAMPs, including circulating nucleic acids/oxidized DNA and advanced glycosylated end products (AGEs). All these materials are likely adsorbed to the bead surface of CytoSorb, either directly or secondarily by their likely affinity to lipid-bilayers of extracellular vesicles, platelets and/or coagulation products, which we also found adherent to the Cytosorb bead surface (Supplementary. Fig. S2). As demonstrated by the elution profile in Supplementary Fig. S3, albumin contents were highest in fractions F19–F24 (Fig. 7a). High DNA contents, determined by NanoDrop assisted spectroscopy, were restricted to F11–F15 and F19–F22, being 1000–4000 ng/ml (Fig. 7b). Fractions F11–F22, F24 and F39 were subjected to ELISA for oxidized nucleotides (specifically for 8-oxo-guanine residues) measurements. Oxidized nucleotides were found at concentration levels of approximately 10^−5^ less intensity when compared to total DNA (Fig. 7b, inserted graph). Using ELISA to detect families of molecular entities binding AGE-specific antibodies, positive results were found in all fractions, peaking between F11–F20, and being highest in F14 (0.95 µg/ml; Fig. 7c). These results are supported by the identification of aggregates stainable with ProteoStat dye and presenting with characteristic morphological features determined by transmission electron microscopy (Supplementary Fig. S5). Further studies on the proteins adsorbed to CytoSorb filters would need to be performed by array formatted assays, including mass spectroscopy (Orbitrap, data analysis in progress). Accordingly, we found proteins related to vesicle trafficking, coagulation and histone modification pathways in F10, F11 and F12, respectively. In F10–F12, we found peptides related to hemoglobin fragments, while the most abundant peptides of F24 were elements from wound healing and endothelial apoptosis processes, in addition to significant amounts of alpha-1-acid glycoprotein 1 and serum amyloid A-1 (SAA1) (data not shown). All these results need to be confirmed by the analysis of fractions isolated from additional hemoadsorption cartridges.

In summary, the molecular entities fractionated from CytoSorb adsorbers of a patient with septic shock had a major impact on mEC confluency, cell layer integrity, proliferation, ATP contents mEC apoptosis. These features may contribute to the clinical observations of perfusion deficiency and organ failure in patients with septic shock.

## Discussion

A major pathology in septic shock occurs by endothelial cell damage, which contributes to organ failure. The pathology related to coagulation factors has been excellently reviewed by Opal and van der Poll^23^. Essentially, soluble factors and activated immune cells contribute to alterations in coagulation and cause increased endothelial permeability. William Aird drewed the following conclusion: “…it would be difficult to identify a single component whose therapeutic modulation will short-circuit the sepsis cascade and improve outcome^34^”, our results provide further evidence for multiple protein entities involved in different pathways to cause sepsis-related endothelial dysfunction. *In vivo*, additional effects are expected to occur by leukocytes interacting with mEC in an inflammatory environment. As reviewed in an excellent manuscript, the co-activation of both cytoplasmic toll-like receptror-(TLR)4 - and TLR3-guided non-canonical inflammasome activation is a likely target to attenuate severity in septic shock models^35^. One reason was the fulminant beneficial effect by CytoSorb hemoadsorption to restore endothelial function and restrict septic shock through a major reduction of catecholamine treatment within 24 hours. We first addressed inflammatory mediators removed by CytoSorb treatment, similar to previous^34^ investigations focusing on inflammatory cytokines (IL-6) and coagulation factors^9,10^, and then fractionated the proteins adsorbed to a CytoSorb column by classic peptide biochemistry. An unbiased approach was followed using a novel bioassay with brain-derived mEC. A total of 39 CytoSorb-derived fractions were tested by long-term video microscopy using an IncuCyteZOOM device. This assay has the advantage of being able to simultaneously analyze^9,10^ cell proliferation, death and morphology^36^. Protein fractions F10–F12 and F24 were identified as having a major effect on the integrity of endothelial-cell layers and mEC viability, proliferation, ATP content and cell death-related mechanisms, including apoptosis. We are currently attempting to identify individual protein fractions affecting mEC from hemoadsorbed material of two additional patients as well as protein fractions pooled from eight other CytoSorb columns. These results will help us identify the protein fractions that affect mEC viability and identify their contents (cf. Fig. 7). In future experiments, we will focus on the identification of the 15 kDa protein species found in F10–F12 (Supplementary Fig. S6).

Previously, several *in vivo* models have been established using animal models to test endothelial-cell function^37^. In the human setting, endothelial-cell dysfunction cannot be readily addressed *in vivo*, but has been implied to occur after experimental LPS dosage^4^. Using *in vitro*-cultured endothelial cells, functional characteristics are more readily addressed by morphological and biochemical parameters. Most frequently, HUVEC have been used^8^. In this context, a highly relevant report by David *et al*. used HUVEC to demonstrate a detrimental effect on endothelial integrity by plasma samples taken before but not after CytoSorb treatment^8^. However, HUVEC models depend on the addition of exogenous growth factors, source and passage number and are limited for the establishment of a reproducible assay format. By contrast, permanent brain-derived mEC are more favorable for reproducible testing, as exemplified in the present report. The human pathology affecting endothelial function has been modelled by other groups using mouse-, rat- and porcine-derived endothelial cells of micro- and macrovascular origin^38–40^. Broad adsorption of sepsis-related PAMP and DAMP molecules performed by Gruda and colleagues favors the most dramatic effects by inflammatory cytokines as well as DAMPS, including High mobility High-Mobility-Group-Protein B1 (HMBG-1), C5a and S100A8^10^.

The results from the 39 fractions of CytoSorb hemoadsorption-derived protein isolates identified the fractions (F10–F12 and F24) as having the greatest impact on mEC. F10–F12 were found to impair proliferation the most (Fig. 2). In hypothesizing which factors were involved, we considered that vascular endothelial growth factor (VEGF) might play a role by increasing vascular permeability, a major parameter for organ dysfunction in septic shock^41^. Increased vascular permeability can also be addressed in our model because this parameter is related to cell death and morphological changes of the mEC monolayer, which may resemble effects by Hcy determined at early time points (Fig. 5). Using apoptosis assays, impaired confluence of *in vitro* cell culture was linked to increased apoptosis, most pronounced for fraction F10. Accordingly, F10 may contain a high amount of VEGF or some as yet undefined effector of vascular permeability. However, VEGF- and VEGF receptor-coordinated endothelial interactions are highly complex and need to be addressed in greater detail^42^, should this factor and its receptor be involved. An important parameter to identify mediators removed by CytoSorb treatment comprises the kinetic observations in our mEC assay. Fractions F10–F12 required more than 10 hours of incubation with mEC to exhibit their specific influence on cell proliferation (Fig. 2a,b), whereas F24 required longer (Fig. 2c). These results argue against the involvement of a highly specific effector in mEC cell damage, which is why homocysteine was considered. When using Hcy as a positive activator of apoptotic cell death in our bioassay (Fig. 3)^29^, we found that Hcy required less than 15 hours for its negative impact on mEC proliferation as well as in inducing apoptosis, whereas later than 15 hours of Hcy treatment, we observed a recovery of the mEC layer (Fig. 4). It is currently unknown whether the homocysteine-specific kinetics of the mEC-layer impairment results from homocysteine degradation or endogenous counter regulatory effects exhibited by mEC. By contrast, mEC incubated with F10–F12, did not appear to recover during our observation times extended to 60 hours (Fig. 2a,b). The prolonged effect on mEC-impaired proliferation and viability may also be explained by declining ATP contents (Fig. 6). Reduced ATP is a hallmark of septic shock and provides evidence for impaired complex I and IV respiration^43^. Nevertheless, the importance of ATP contents in leukocytes of patients with sepsis and septic shock is still greatly discussed^43^. Moreover, reduced ATP may result from increased nitrosylation of the electron transport-chain complexes occurring in endothelial cells when endothelial nitric oxide synthase (eNOS) is impaired and inducible nitric oxide synthase (iNOS) is upregulated^44^. Similarly, the dysbalance between eNOS and iNOS induced by inflammatory stimuli is a well-accepted biomarker for inflamed microvascular endothelial cells^34^. SAA1 is one of the most common acute-phase proteins. It has been described that SAA1 can interact with TLRs and RAGE^45^, activating NFκB and promoting cytokine production^46^. In a mouse model, low SAA1 improved the outcome by attenuating HMGB1 release^45^. A direct interaction of SAA1 with endothelial cells induces ROS release and would impair beneficial eNOS activity^47^. In an animal sepsis model, the selective contribution of aging to upregulated inflammation by oxidative stress and concomitant mitochondrial dysfunction were demonstrated^48^. These parameters, that is, oxidative stress and mitochondrial dysfunction, causes endothelial dysfunction and would be followed by organ dysfunction and high mortality in sepsis^48^. The potential relevance of oxidized and in-part denatured molecular entities has been further substantiated by our study. By ELISA-based quantification, we found high levels of oxidized DNA, enriched in F11–F15 (Fig. 7). In analogy to the properties described for CytoSorb hemoadsorption^10^, we found DAMP elements, including AGEs, in multiple fractions of the CytoSorb-hemoadsorbed protein isolates. The highest concentrations were present in F14–F20, and not in F10–F12, or F24. One property of DAMPs is impaired degradation *in vivo* because of insoluble beta sheet-rich aggregates, as found in F21 and F36, which stain with ProteoStat dye (Supplementary Fig. S5). Importantly, such aggregates function as potent activators of the inflammasome, acting as triggering factors for NACHT, LRR and PYD domains-containing protein 3(NLRP-3), culminating in inflammation-associated degeneration^49^. Both oxidized DNA and AGEs would then bind to the receptor of AGEs, named RAGE, a protein which is highly expressed on the endothelium, smooth muscle cells and macrophages^50^. RAGE interaction with its ligands are expected to lead to massive activation of the inflammasome^50,51^. These processes may best explain the morphological changes and monolayer disruption as well as the formation of stress-associated microvilli (Fig. 5) in our mEC. Xi *et al*. previously demonstrated that inflammasome activation may occur by Hcy and simultaneously result in endothelial dysfunction^28^.

In summary, the current work describes a mEC-based bioassay to study blood-derived mediators, which were efficiently eliminated by CytoSorb hemoadsorption treatment and may explain rapid resolution from states of septic shock after CytoSorb treatment. The results provide evidence for soluble plasma-derived factors with multiple effects on endothelial-cell barrier function and viability as well as ATP contents. Preliminary results imply the contribution of different players from coagulation signaling pathways, vesicle trafficking and/or histone modification, which were identified in fractions from hemoadsorption purifications that impaired endothelial viability. Further efforts are needed to address the molecular nature of the most detrimental factor combinations.

## Conclusion

Detrimental effects of CytoSorb-bound hemoadsorption compounds on mEC using our mEC based bioassay have been identified after biochemical enrichment. These compounds were in part characterized by their contents of oxidized nucleic acids and glycosylation end products. The functional impairments of mEC layers were (i) delayed proliferation and confluence, which might correlate with decreased tissue repair; (ii) increased cell death and apoptosis, correlating to an immediate defect of organ protection; and (iii) changes in intracellular ATP contents to explain various dysfunctional aspects of the endothelium in cell recruitment and leukocyte interactions. Future plans will focus on sub fractionation experiments and the molecular identification of 2–3 major fractions with the highest biological activities. The results are expected to better characterize the molecular and functional aspects in reductions of endothelial damage and clinical remission states induced by CytoSorb hemoadsorption procedures.

## Supplementary information

Supplementary Figure S1-S6

Supplementary video 

## Data Availability

The datasets generated during and/or analyzed during the current study are available from the corresponding author on reasonable request.
