# Supplementary material for: Bioassay for Endothelial Damage Mediators Retrieved by Hemoadsorption
Source: Sci Rep. 2019 Oct 10;9:14522. doi: 10.1038/s41598-019-50517-1 (PMC6787199; doi:10.1038/s41598-019-50517-1)
Supplement: Supplementary file 1 — Supplementary Figure S1-S6 [file 41598_2019_50517_MOESM1_ESM.pdf]

Bioassay for Endothelial Damage Mediators Retrieved by Hemoadsorption

Maximilian Denzinger[1], Ludger Staendker[2], Keno Ehlers[1], Julian M. Schneider[1], Tanja Schulz[1], Tabea Hein[1], Sebastian Wiese[3], Annika Roecker[4], Ruediger Gross[4], Jan Münch[2,4], Hendrik Bracht[5], Eberhard Barth[5], Manfred Weiss[5], Michael Georgieff[5], E. Marion Schneider[1]

[1]Division of Experimental Anesthesiology, University Hospital Ulm, Albert-Einstein-Allee 23; 89081 Ulm, Germany; [2]Core Facility Functional Peptidomics, Ulm University, Albert-Einstein-Allee 47; 89081 Ulm, Germany; [3]Institute of Pharmacology and Toxicology, Ulm University, Albert-Einstein-Allee 11, 89081 Ulm, Germany; [4]Institute of Molecular Virology, Ulm University Medical Center, Meyerhofstrasse 1, 89081 Ulm, Germany; [5]Department of Anesthesiology, University Hospital Ulm, Albert-Einstein-Allee 23; 89081 Ulm, Germany.

Supplementary Figure S1

a **b**

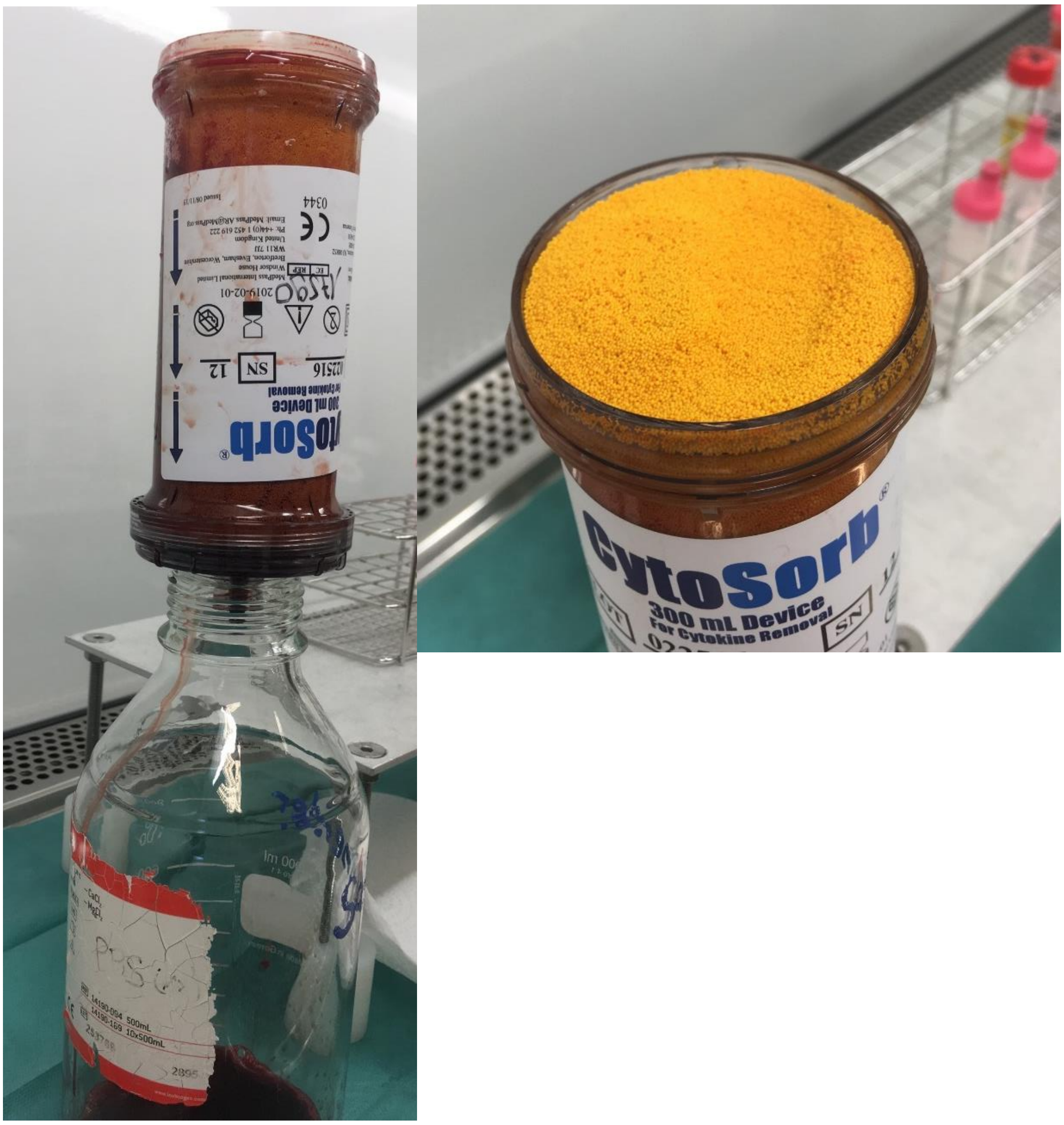

Washing procedure of hemoadsorption cartridges. Following disconnection from the extra-corporal circulation, the cartridge is opened at both ends, the inner filter membrane is removed from the upper end and the cartridge is placed on top of an empty autoclaved glass bottle following the direction of the extra-corporal flow **(a).** The cartridge is washed with phosphate-buffered saline, 0.005% ethylenediaminetetraacetic acid (EDTA) until the outflow is clear from blood cells (approximately 500 ml washing solution are required for this procedure). Cartridge beads become colored from plasma components, including bilirubin among others **(b).**

Supplementary Figure S2

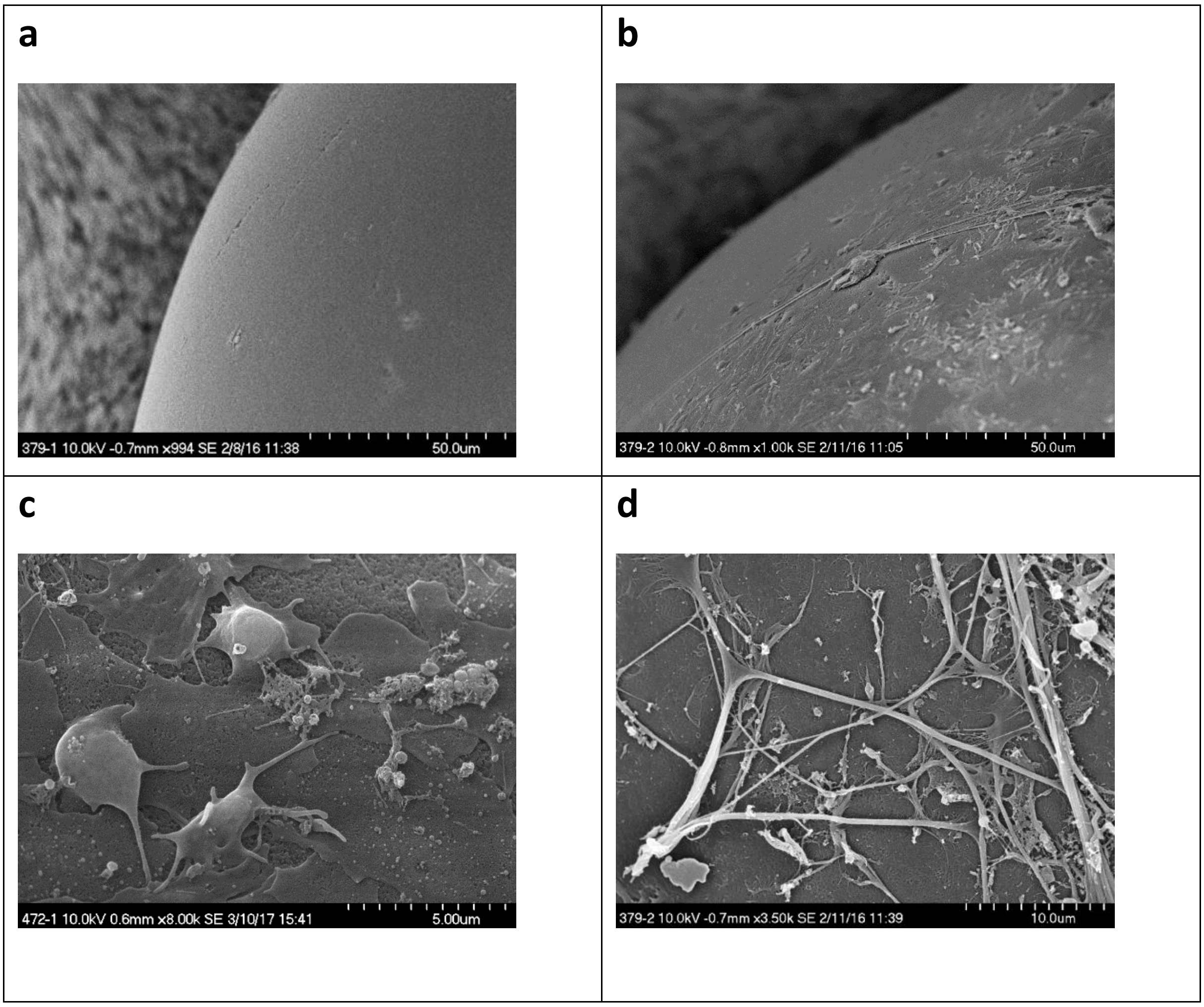

Scanning electron micrographs of the surface of Cytosorb® beads. Unused bead surface **(a)** and a used bead surface with protein material attached (**b**), at low magnification. At higher magnification for the used beads, adherent protein material and some platelets can be distinguished **(c)** as well as clotting proteins **(d)**.

Supplementary Figure S3

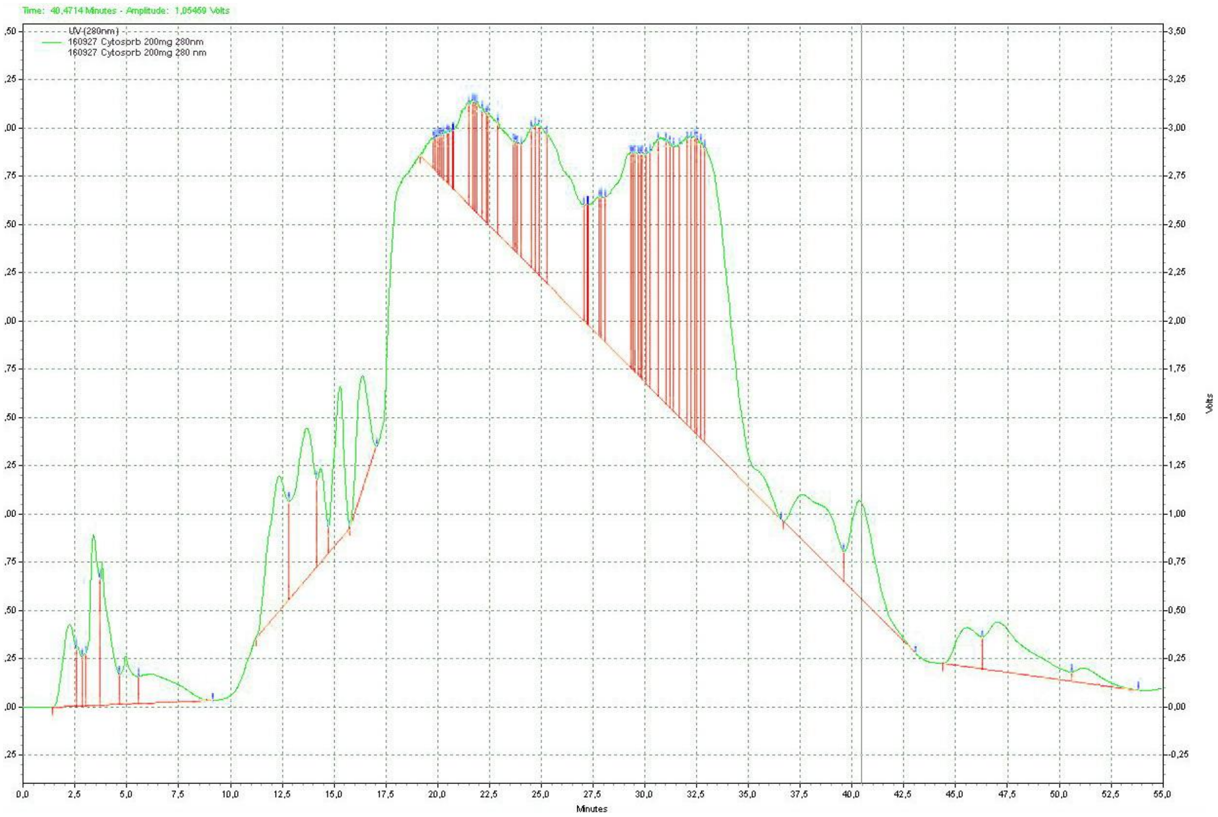

Protein fractionation procedure of Cytosorb material. Protein detachment from Cytosorb beads was performed using 50% acetonitrile in water for 1 hour, 1/10 dilution of the clear extract and a subsequent ultrafiltration step (cut-off: 30 kDa) to remove albumin. The peptides were then separated by standard reverse phase chromatography using a linear acetonitril gradient. Fractionation time is given on the x-axis, protein content was determined by 280/260 nm adsorption and is shown on the y-axis; red vertical lines indicate fractions.

Supplementary Figure S4

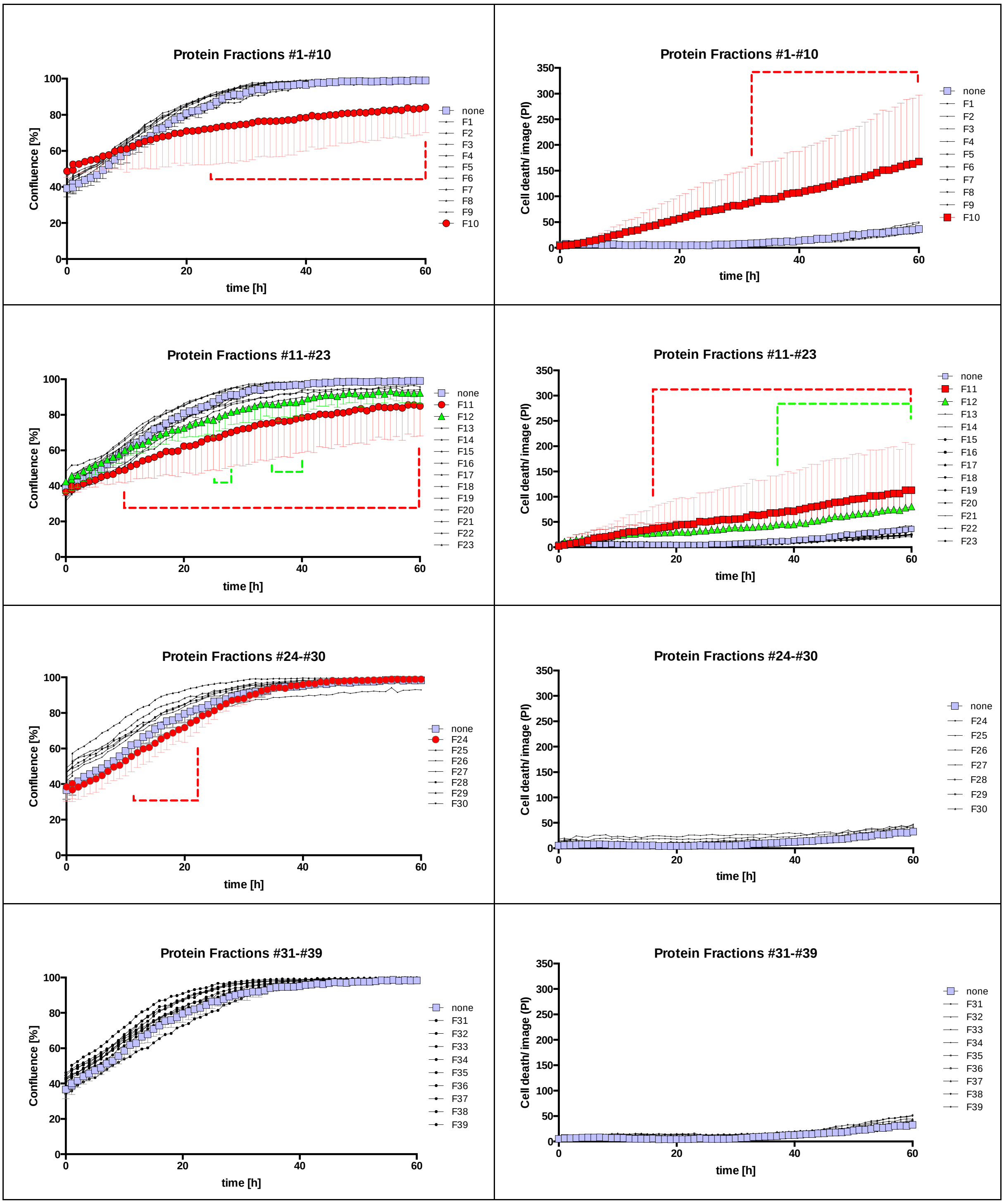

Effects of Cytosorb protein fractions on microvascular endothelial cells (mEC) growth and cell death. MEC were cultured in the absence or presence of 1:10 diluted protein fractions (#1-#39) for up to 60 hours (x-axis). Confluency was determined by optical analysis using the IncuCyteZOOM. Protein fractions resulting in significantly diminished confluence rates are shown as red symbols following two-way ANOVA (multiple comparison) analysis. Protein

fractions resulting in cell death of cultured mEC were determined by adding 2 ng/ml propidium iodide to the culture medium; counting of red fluorescent nuclei was accomplished by IncuCyteZOOM fluorescence video microscopy and red object count quantification. Fractions leading to significantly increased cell death are shown by red- or green-filled symbols. Significances were determined by two-way ANOVA (multiple comparison) analysis. All values are given as means and standard deviation of triplicate cultures for statistically relevant protein fractions. Images were obtained with the IncuCyteZOOM using a 20× objective, phase contrast and fluorescence detection (565–605 nm excitation, 625–705 nm emission).

Supplementary Figure S5

a

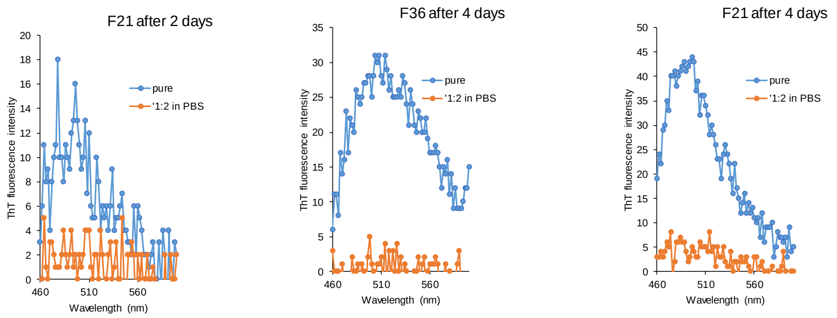

Protein fractions F21 and F36 potentially contain amyloid

b

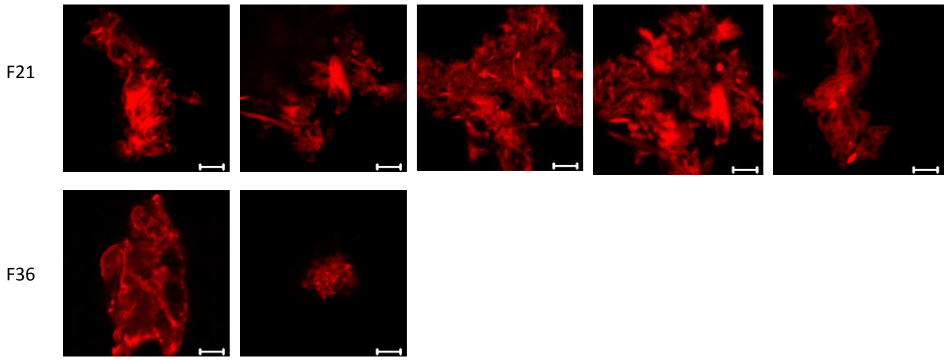

Insoluble β-sheet formation from protein fractions F21 and F36

c

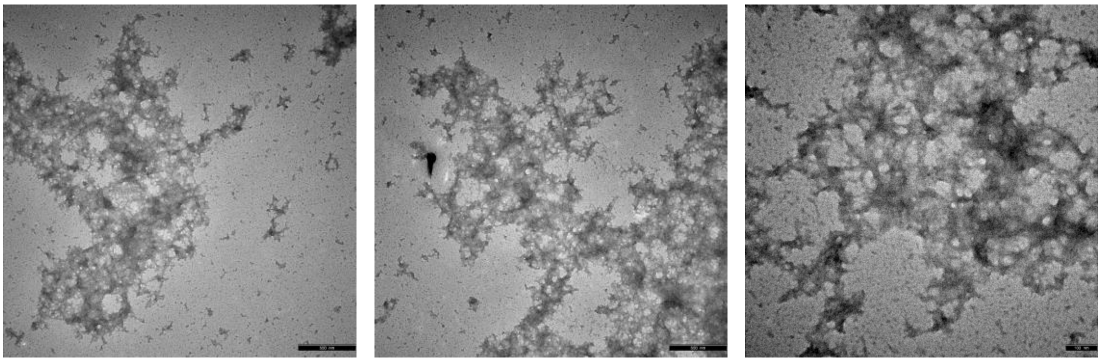

Transmission electron micrographs of F21

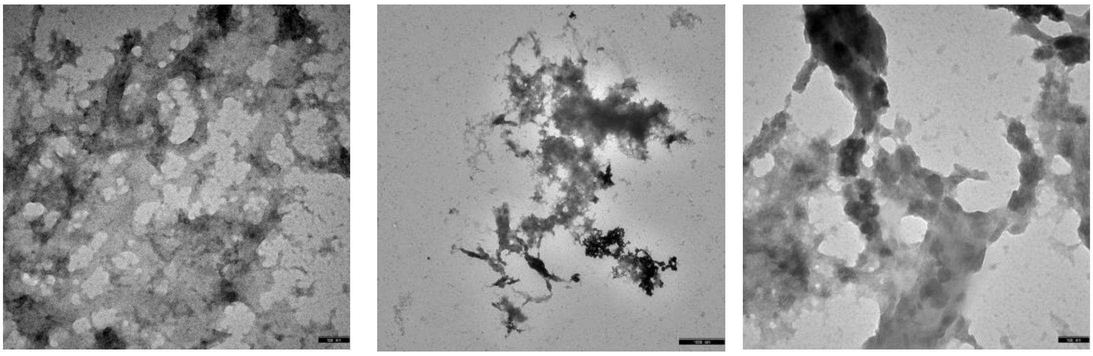

Transmission electron micrographs of F36

Amyloid-detection assays for Cytosorb-derived protein fractions. All fractions (F1–F39) were structurally analyzed for the potential presence of insoluble beta sheet-rich aggregates, including amyloid-forming proteins. Insoluble aggregates were positive in fractions F21 and F36 after Proteostat staining using the Aggresome detection kit, (pFTAA), (enzolifesciences.com). Fluorescence intensities were further quantified by analyzing regions of interest and are shown as arbitrary fluorescence units with emission wavelengths of 460–600 nm, either non-diluted (blue lines) or 1:2 dilution in phosphate-buffered saline (orange lines) (**a**). Aggresome formation was tested after 2 and 4 days of incubation at 37°C and centrifugation at 1400 rpm. After 2 days at 37°C and 1400 rpm centrifugation, all samples from the Cytosorb fractions were non-turbid (**b**). F21 and F36 gave a positive fluorescence signal (but were negative in the pFTAA, aggresome detection test) while the remaining fractions were negative both for fluorescence and pFTAA. Transmission electron micrographs (TEM) of the aggresome formation assay provide further proof for insoluble β-sheet formation in fraction F21 and to a lesser extent in fraction F36 **(c)**. Bars are equivalent to 5 µm.

Supplementary Figure S6

a **b**

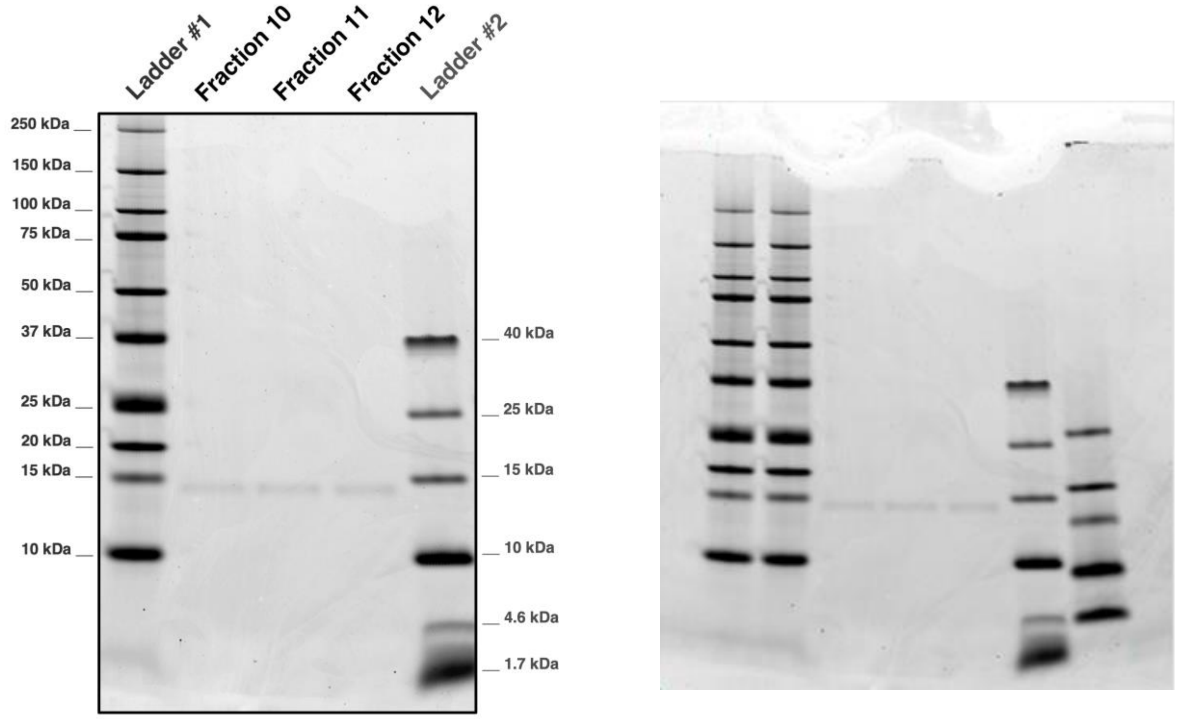

Molecular weight analysis of the protein contents in F10–F12. SDS-PAGE of Cytosorb-derived peptide fractions F10–F12. Aliquots of each peptide fraction were resuspended in 1x Novex Tricine SDS sample buffer (thermofisher.com), containing 50 mM TCEP (Tris(2-carboxyethyl)phosphine) as a reducing agent. Samples were heated to 85°C for 2 minutes, loaded onto a Novex 10–20% Tricine protein gel (thermofisher.com) alongside size standards (ladder #1: Precision Plus Kaleidoscope, biorad.com; Ladder #2: Specta Multicolor Low Range, thermofisher.com) and ran at 125V for 90 minutes. The SDS-PAGE gel stained with GelCode Blue Coomassie (thermofisher.com) was scanned on a LiCor Near-Infrared Imager. The 700 nm channel was set to 7.5 intensity and 169 µm resolution. The final figure (**a**) was derived from image (**b)** by cropping, and encompassing the entire region from 1.7 to 250 kDa. Two different protein ladders were used for comigration.
